# Association of Serum Anti-PCSK9 Antibody Levels with Favorable Postoperative Prognosis in Esophageal Cancer

**DOI:** 10.3389/fonc.2021.708039

**Published:** 2021-08-24

**Authors:** Masaaki Ito, Takaki Hiwasa, Yoko Oshima, Satoshi Yajima, Takashi Suzuki, Tatsuki Nanami, Makoto Sumazaki, Fumiaki Shiratori, Kimihiko Funahashi, Shu-Yang Li, Yasuo Iwadate, Hiroki Yamagata, Byambasteren Jambaljav, Minoru Takemoto, Koutaro Yokote, Hirotaka Takizawa, Hideaki Shimada

**Affiliations:** ^1^Department of Clinical Oncology, Toho University Graduate School of Medicine, Tokyo, Japan; ^2^Department of Neurological Surgery, Chiba University Graduate School of Medicine, Chiba, Japan; ^3^Department of Gastroenterological Surgery, Toho University School of Medicine, Tokyo, Japan; ^4^Department of Diabetes, Metabolism and Endocrinology, School of Medicine, International University of Health and Welfare, Chiba, Japan; ^5^Department of Endocrinology, Hematology and Gerontology, Graduate School of Medicine, Chiba University, Chiba, Japan; ^6^Port Square Kashiwado Clinic, Kashiwado Memorial Foundation, Chiba, Japan

**Keywords:** proprotein convertase subtilisin/kexin type 9, programmed cell death ligand 1, esophageal cancer, antibody biomarker, overall survival, hyperlipidemia

## Abstract

**Background:**

Esophageal cancer often appears as postoperative metastasis or recurrence after radical surgery. Although we had previously reported that serum programmed cell death ligand 1 (PD-L1) level correlated with the prognosis of esophageal cancer, further novel biomarkers are required for more precise prediction of the prognosis. Proprotein convertase subtilisin/kexin type 9 (PCSK9) is associated with the cholesterol metabolism. But there was no report of relationship between serum PCSK9 antibody and cancer. Therefore, we investigated whether anti-PCSK9 antibodies could be a novel biomarker for solid cancer.

**Methods:**

Serum levels of anti-PCSK9 antibodies and antigens in patients with solid cancer were analyzed using amplified luminescence proximity homogeneous assay-linked immunosorbent assay (AlphaLISA). The reactivity of serum antibodies against recombinant PCSK9 protein was investigated by Western blotting, and the expression of PCSK9 antigens in esophageal cancer tissues was examined by immunohistochemical staining.

**Results:**

AlphaLISA showed that serum anti-PCSK9 antibody (s-PCSK9-Ab) levels were significantly higher in patients with esophageal cancer, gastric cancer, colorectal cancer, lung cancer, and breast cancer than in healthy donors, and patients with esophageal cancer had the highest levels. The presence of serum antibody in patients was confirmed by Western blotting. There was no apparent correlation between s-PCSK9-Ab and PCSK9 antigen levels. Immunohistochemical staining demonstrated the expression of PCSK9 antigen in both the cytoplasm and nuclear compartments of esophageal squamous cell carcinoma tissue but not in normal tissue. Compared with patients with low s-PCSK9-Ab levels, those with high s-PCSK9-Ab levels had a favorable postoperative prognosis after radical surgery for esophageal cancer. In the multivariate analysis, tumor depth and s-PCSK9-Ab level were identified as independent prognostic factors. In the univariate analysis of clinicopathological features, high PCSK9 antibody levels were not associated with sex, age, location, tumor depth, lymph node status, squamous cell carcinoma antigen, or p53-Ab, whereas they correlated significantly with PD-L1 levels, which were associated with unfavorable prognosis. Correlation between s-PCSK9-Ab and PD-L1 levels was also confirmed in the logistic regression analysis; therefore, low s-PCSK9-Ab levels could discriminate another poor prognosis group other than high-PD-L1 group.

**Conclusions:**

Patients with solid cancer had higher s-PCSK9-Ab levels than healthy donors. High s-PCSK9-Ab levels indicated better prognosis for overall survival after surgery in patients with esophageal cancer.

## Introduction

Esophageal cancer progresses rapidly and has a very poor prognosis compared with other solid cancers (1). There are several cases of metastatic recurrence, and hence the malignant potential is high even if it receives adequate therapy in the early stage (2). Various anticancer drugs have been recently developed for cancers of digestive organs, including immune checkpoint inhibitors. Nivolumab has been recently used for treating esophageal cancer worldwide; however, the treatment results have not been necessarily satisfactory (3). Regarding newly developed biomarkers, we had previously reported that high serum levels of programmed cell death ligand 1 (PD-L1) exhibited poor prognosis in patients with esophageal cancer (4). PD-L1 could become a predictive marker of prognosis, and it is expected to be used in the early diagnosis and treatment of immune checkpoint inhibitors for improving curability.

The proprotein convertase subtilisin kexin/type 9 (PCSK9) gene mutations was first identified to cause autosomal dominant hypercholesterolemia for a risk factor of coronary heart disease (5). PCSK9 is affected to cholesterol metabolism *via* low density lipoprotein receptor destruction (6–9). And PCSK9 inhibition can be used for treatment of hypercholesterolemia (10). The United States Food and Drug Administration already approved two monoclonal antibodies (evolocumab, alirocumab) to treat hypercholesterolemia. Recently Large-scale clinical randomized trials using PCSK9 monoclonal antibody were performed. ODYSSEY investigators conducted a randomized trial involving for 2341 patients and demonstrated that alirocumab showed significantly reduced LDL cholesterol levels (11). In addition, the risk of recurrent ischemic cardiovascular events for alirocmab after acute coronary syndrome patients was reduced (12). In terms of evolocumab, a randomized, double-blind, placebo-controlled FOURIER Clinical Trial was conducted with 27,564 patients (13). And the results also showed that inhibition of PCSK9 lowered LDL cholesterol levels and reduced the risk of cardiovascular events. It was still controversial whether serum cholesterol influenced to cancer (14–18), but in terms of PCSK9 and cancer several reported were documented (19, 20).

Recently, Liu et al. reported that deleting the PCSK9 gene in mouse cancer cells substantially attenuated or prevented cancer growth in mice in a manner that depended on cytotoxic T cells. It also enhanced the efficacy of immune therapy that was targeted at the checkpoint protein PD1. Moreover, clinically approved PCSK9-neutralizing antibodies were found to synergize with anti-PD1 therapy in suppressing tumor growth in mouse models of cancer (21). Besides, PCSK9 is thought to be engaged in multiple biological processes including cell cycle, inflammation, and apoptosis (22–27). But there was no report concerned to the relationship between serum PCSK9 autoantibody and cancer. Therefore, in the present study, we investigated the levels of serum anti-PCSK9 autoantibodies (s-PCSK9-Ab) and serum PCSK9 antigen (s-PCSK9-Ag) in patients with solid cancer, as well as their clinicopathological features and prognosis.

## Materials and Methods

### Collection of Serum Samples

Serum samples from patients with various types of cancer involving the esophagus (n = 192), stomach (n = 96), colorectum (n = 192), lung (n = 96), and breast (n = 96) were obtained. A total of 96 healthy donor (HD) samples were collected from Port Square Kashiwado Clinic.

Among the 192 patients with esophageal cancer, 91 underwent radical surgery at Toho University Omori Hospital between June 2010 and February 2016. A total of 63 of these patients received neoadjuvant chemotherapy. The number of patients in each stage (Japanese Classification of Esophageal Cancer, 11th Edition) (28) was as follows: 9 patients in stage 0, 14 patients in stage I, 25 patients in stage II, 34 patients in stage III, and 9 patients in stage IVa. All patients were followed up till July 2018 or death. Each clinicopathological feature and prognosis of these patients was examined. The cutoff level of serum PD-L1 was set at 75 percentile (65.6 pg/mL) of esophageal cancer (4).

### Measurement of s-PCSK9-Ab and -Ag Levels and Conventional Serum Markers

Serum samples were collected before treatment, centrifuged at 3000 *g* for 10 min, and stored at −80°C until use. The levels of s-PCSK9-Ab and -Ag were measured using amplified luminescence proximity homogeneous assay-linked immunosorbent assay (AlphaLISA) for PCSK9. AlphaLISA was conducted using 384-well microtiter plates (white opaque OptiPlate™, PerkinElmer) containing 2.5 μL of 1/100-diluted sera and 2.5 μL of GST or GST-fusion proteins (10 μg/mL) in AlphaLISA buffer (25 mM HEPES, pH 7.4, 0.1% casein, 0.5% Triton X-100, 1 mg/mL dextran-500, and 0.05% Proclin-300) according to the manufacturer’s instructions (PerkinElmer, http://www.perkinelmer.com/lab-solutions/resources/docs/GDE_ELISA-to-AlphaLISA.pdf). The reaction mixture was incubated at room temperature for 6–8 h, after which anti-human IgG-conjugated acceptor beads (2.5 μL of 40 μg/mL) and glutathione-conjugated donor beads (2.5 μL of 40 μg/mL) were added and incubated further for 7–21 days at room temperature in the dark. The chemical emission at 607–623 nm (Alpha photon count) which represents the antigen-antibody binding level was read on an EnSpire Alpha microplate reader (PerkinElmer) as described previously (29–31). Specific reactions were estimated by subtracting the Alpha values of GST control from the values of GST-fusion proteins. The levels of serum p53 antibodies (p53-Abs) (32) and squamous cell carcinoma antigen (SCC-Ag) (33) were also evaluated as previously described. The cutoff values for serum p53-Abs and SCC-Ag were set at 1.3 IU/mL and 1.5 ng/mL, respectively.

### Purification of Recombinant Proteins

The full-length coding sequence of human PCSK9 gene (NCBI Accession number: NM_174936.3) was recombined into *Eco*RI/*Xho*I site of pGEX-4T-1. ECOS™ competent *Escherichia coli* JM-109 cells (Nippon Gene) were transformed with the eukaryotic expression plasmid pGEX-4T-1 or pGEX-4T-1-PCSK9 and then cultured for 3 h in 200 mL of Luria broth (LB) containing 0.1 mM isopropyl β-d-thiogalactopyranoside (IPTG; Wako Pure Chemicals, Osaka, Japan). Next, the cells were harvested, washed with phosphate-buffered saline, and lysed by sonication in BugBuster Protein Extraction Reagent (Merck Millipore, Darmstadt, Germany). Lysates were centrifuged at 15,000 *g* for 10 min at 4°C, and GST and GST-fused PCSK9 proteins were purified by affinity chromatography using glutathione–Sepharose columns (GE Healthcare Life Sciences) as described previously (34–36).

### Western Blotting

GST and GST-fused PCSK9 proteins (0.3 μg) were separated on sodium dodecyl sulfate-polyacrylamide gels. After transfer, the membranes were incubated with anti-GST antibodies (Rockland, Gilbertsville, PA), anti-PCSK9 antibody (GeneTex, CA), or sera from HDs or patients with esophageal cancer. After incubation with a horseradish peroxidase-conjugated secondary antibody, immunoreactivity was detected using Immobilon (Merck Millipore, Darmstadt, Germany) as described previously (29, 35–38).

### Immunohistochemical Staining

Formalin-fixed paraffin-embedded esophageal cancer tissues were cut into 4-µm-thick sections. Sections were deparaffinized, blocked with a detection kit (ab64261, Abcam, Cambridge, UK), reacted with primary anti-PCSK9 antibodies (GTX81524, rabbit polyclonal antibodies, GeneTex, CA) at 2 µg/mL for 1 h at room temperature, incubated with biotinylated anti-goat IgG, and reacted with streptavidin conjugated to horseradish peroxidase reagent. Finally, the reaction was visualized using a diaminobenzidine chromogen. Sections were then counterstained with hematoxylin, dehydrated, and mounted.

### Statistical Analysis

The Mann–Whitney *U* test was used for comparisons between unpaired groups. Differences in the distribution of two variables were evaluated using Fisher’s exact test. The Kruskal-Wallis test (Mann–Whitney *U* test with Bonferroni’s correction applied) was used to evaluate the corresponding differences among three variables. Clinicopathological data were analyzed using logistic regression analysis to evaluate the association with serum PCSK9 antibody level. Survival curves were plotted using the Kaplan–Meier method and compared using log-rank test. The Cox proportional hazards model was used to evaluate significant predictors. All analyses were conducted using the EZR software (39). Statistical significance levels were defined as p <0.05, written bold in tables.

## Results

### Comparison of S-PCSK9-Ab and S-PCSK9-Ag Levels Between HDs and Patients With Solid Tumors

**Figure 1:**
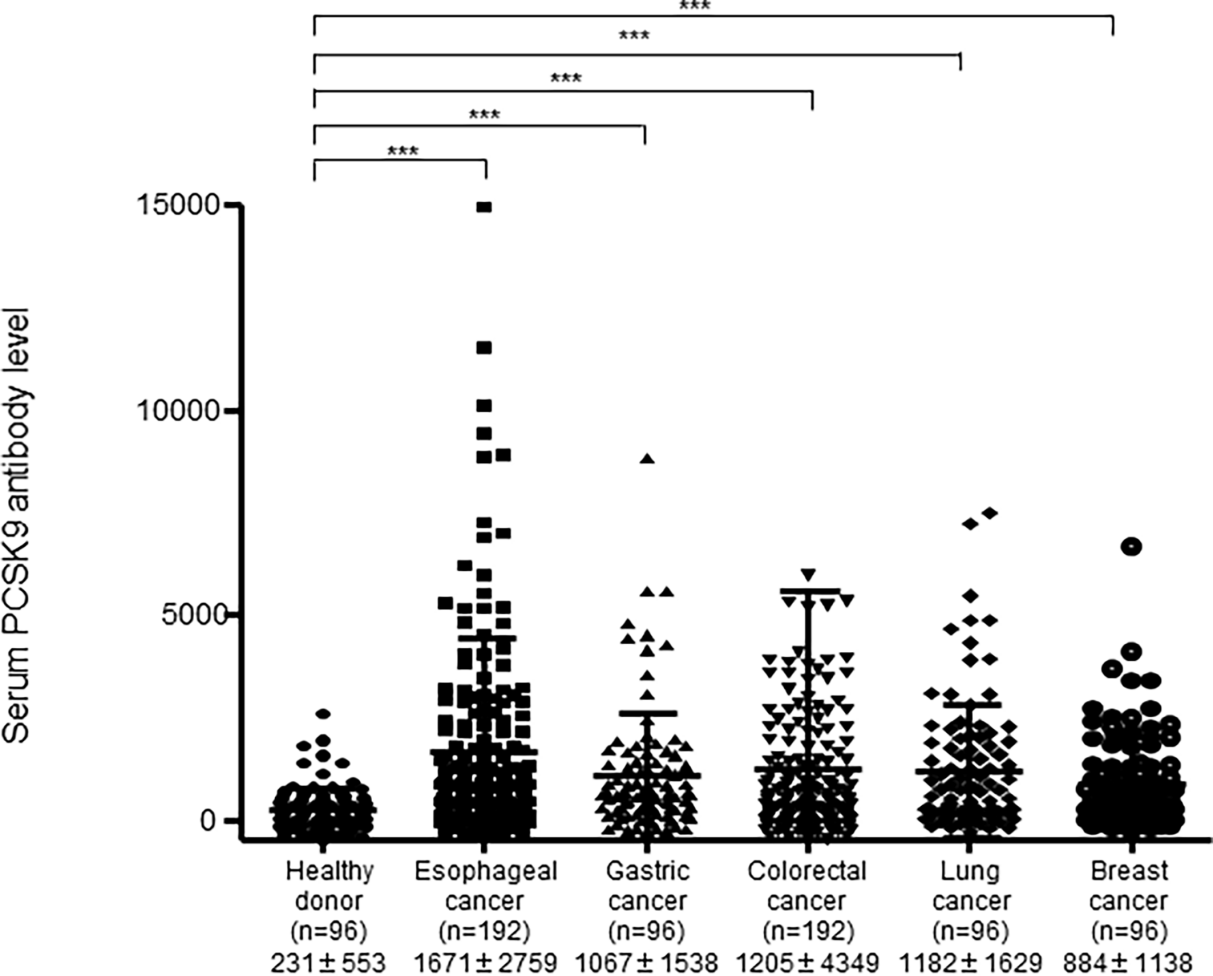
Comparison of serum anti-PCSK9 antibody (s-PCSK9-Ab) levels between solid cancers and healthy donors. The levels of s-PCSK9-Abs in HDs and patients with esophageal cancer, gastric cancer, colorectal cancer, lung cancer, and breast cancer examined by AlphaLISA are shown. The bars represent mean and mean ± SD. The numbers in parentheses indicate the number of cases. Arabic numerals represent PCSK9 antibody titers. ***p < 0.001; evaluated with the Kruskal-Wallis test.

We recombined the full-length PCSK9 cDNA into pGEX-4T-1, purified the GST-PCSK9 protein, and examined the s-PCSK9-Ab levels in patients with esophageal cancer, gastric cancer, colorectal cancer, lung cancer, and breast cancer. The mean s-PCSK9-Ab levels (± standard deviation; no units) were as follows: HDs, 231 ± 553; patients with esophageal cancer, 1671 ± 2759; patients with gastric cancer, 1067 ± 1538; patients with colorectal cancer, 1205 ± 4349; patients with lung cancer, 1182 ± 1629; and patients with breast cancer, 884 ± 1138. The Kruskal-Wallis test revealed that patients with any type of cancer had significantly higher levels of s-PCSK9-Abs than HDs (**Figure 1**), suggesting that s-PCSK9-Ab is a common marker for solid cancers. The levels were especially higher in those with esophageal cancer and relatively lower in patients with breast cancer.

**Figure 2:**
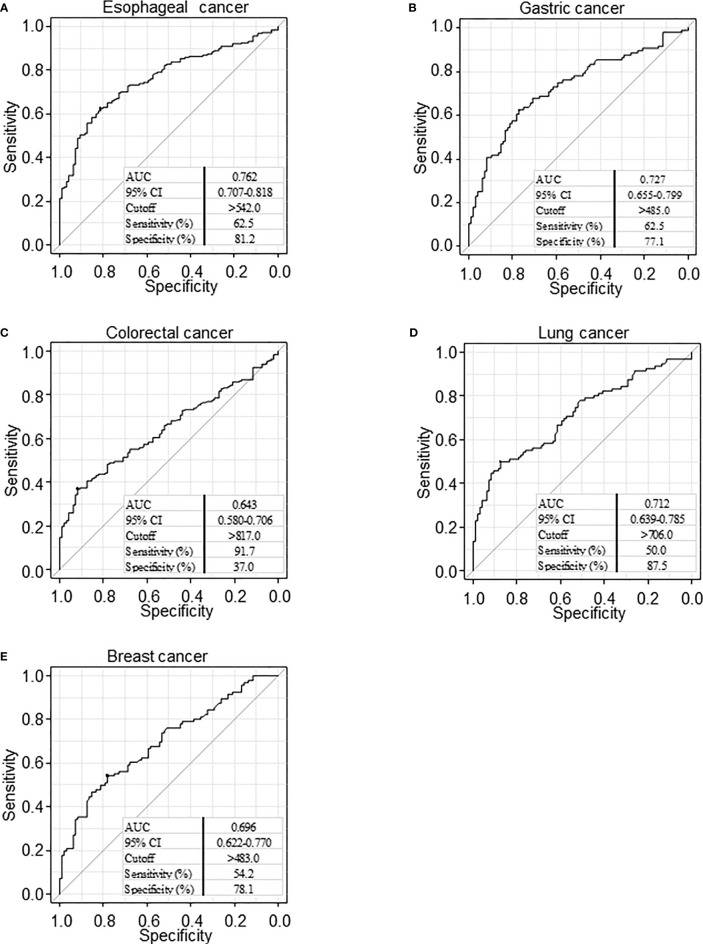
Receiver operating characteristic (ROC) curve analysis. ROC analysis was performed to evaluate sensitivity and specificity between esophageal cancer **(A)**, gastric cancer **(B)**, colorectal cancer **(C)**, lung cancer **(D)**, and breast cancer **(E)** and healthy donor. Numbers in the figure represent cutoff level, specificity and sensitivity. The area below the curve in the graph shows the Area Under the Curve (AUC). AUC takes a value from 0 to 1, and the closer the value is to 1, the higher the discriminant ability. CI, confidence interval.

We then conducted a receiver operating characteristic (ROC) curve analysis to evaluate the sensitivity and specificity between patients with any type of cancer and HDs (**Figure 2**). Results revealed that the area under the curve (AUC) was >0.6 for all cancers. When the cutoff value was determined to be 542, the sensitivity and specificity of serum PCSK9-Abs for patients with esophageal cancer were 62.5% and 81.2%, respectively (**Figure 2A**).

### Western Blotting

**Figure 3:**
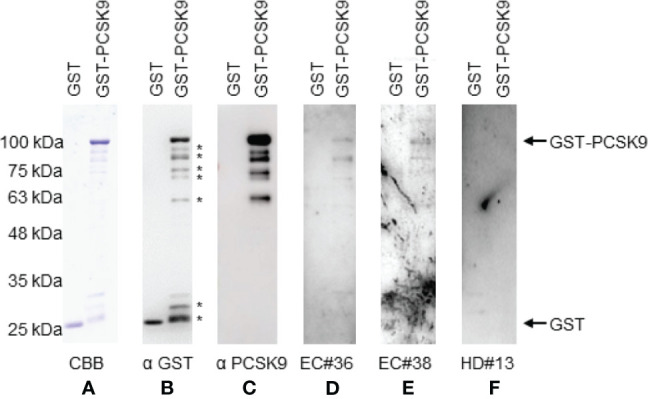
Western blotting analysis of sera with esophageal cancer patient. Representative results of Western blotting are shown. GST and GST-PCSK9 proteins were electrophoresed through SDS-polyacrylamide gels followed by staining with Coomassie Brilliant Blue (CBB) **(A)**, or Western blotting using anti-GST (αGST) **(B)**, anti-PCSK9 antibody (αPCSK9) **(C)**, sera of patients [#36 **(D)**, #38 **(E)**], or a healthy donor serum [#13 **(F)**]. The arrows indicate the positions of GST-PCSK9 and GST. The asterisks represent degradation products of GST-PCSK9. Molecular weights are shown to the left.

The presence of anti-PCSK9 antibodies in patients’ sera was confirmed by Western blotting. GST-PCSK9 and GST proteins were detected as 100- and 26-kDa proteins, respectively, using the anti-GST antibody (**Figures 3A, B**). The asterisks in the figure represent the degradation products. GST-PCSK9 but not GST reacted with the PCSK9 antibody (**Figure 3C**). GST-PCSK9 was recognized by serum IgG antibodies of patients with esophageal cancer (EC#36, EC#38) but not by those of HDs (**Figures 3D–F**). The GST protein exhibited no apparent reactivity against serum IgG antibodies, irrespective of those obtained from HDs or patients with esophageal cancer.

### Comparison of s-PCSK9-Ag Levels Between HDs and Patients With Esophageal and Gastric Cancer

**Figure 4:**
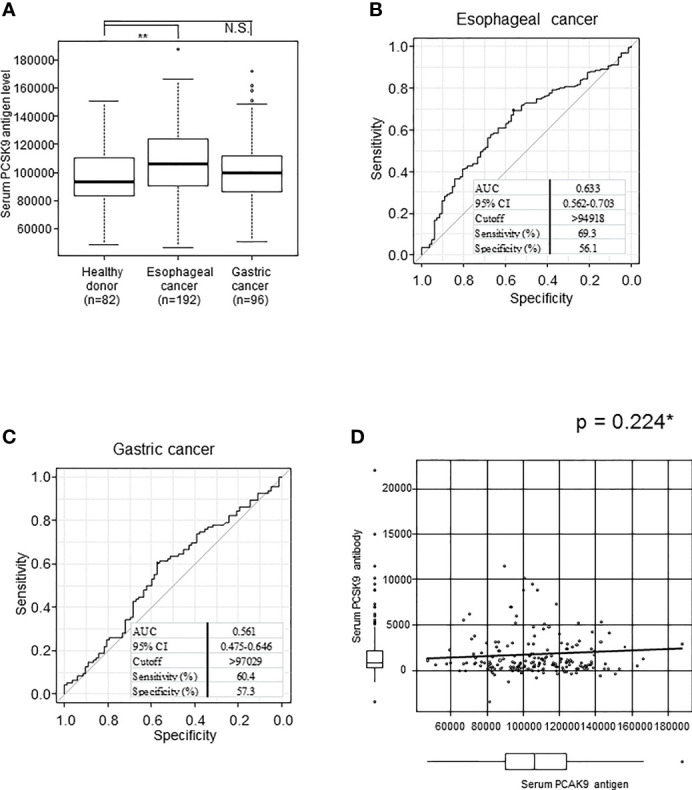
Comparison of s-PCSK9-Ag levels between esophageal cancer, gastric cancer and healthy donors. The levels of serum PCSK9 in HDs and patients with esophageal cancer and gastric cancer examined by AlphaLISA are shown in a box-whisker plot **(A)**. The bars represent mean and mean ± SD. **p < 0.01, evaluated with the Kruskal-Wallis test. NS, not significant. ROC curve analysis was performed to evaluate sensitivity and specificity between each cancer and healthy donor. Numbers in the figure represent cutoff level, specificity and sensitivity **(B, C)**. The correlation between serum PCSK9 antigen and antibody level is shown in a scatter plot **(D)**. *p value was calculated with Spearman’s rank correlation analysis.

We next investigated serum PCSK9 antigen levels in patients with esophageal cancer and gastric cancer and HDs using the same abovementioned methods. The mean s-PCSK9-Ag levels (± standard deviation; SD) were as follows: HDs, 97145 ± 20673; patients with esophageal cancer, 107136 ± 24013; and patients with gastric cancer, 101045 ± 22184. Patients with esophageal cancer had significantly higher levels of s-PCSK9-Ag than HDs (p = 0.002), but no significant difference was observed between patients with gastric cancer and HDs (p = 0.057) (**Figure 4A**). The ROC curve analysis showed that the AUC values of s-PCSK-Ag between patients with esophageal cancer and those with gastric cancer were 0.633 and 0.561, respectively (**Figures 4B, C**).

We then explored the relationship between s-PCSK9-Ab and s-PCSK9-Ag levels. Unexpectedly, no apparent correlation was detected between s-PCSK9-Ab and s-PCSK9-Ag levels (**Figure 4D**), suggesting that the increase in s-PCSK9-Ag levels is not the primary cause for the increase in s-PCSK9-Ab levels.

### Comparison of Serum PCSK9 Antibody and Antigen Levels According to the Pathological Stage of Esophageal Cancer

**Figure 5:**
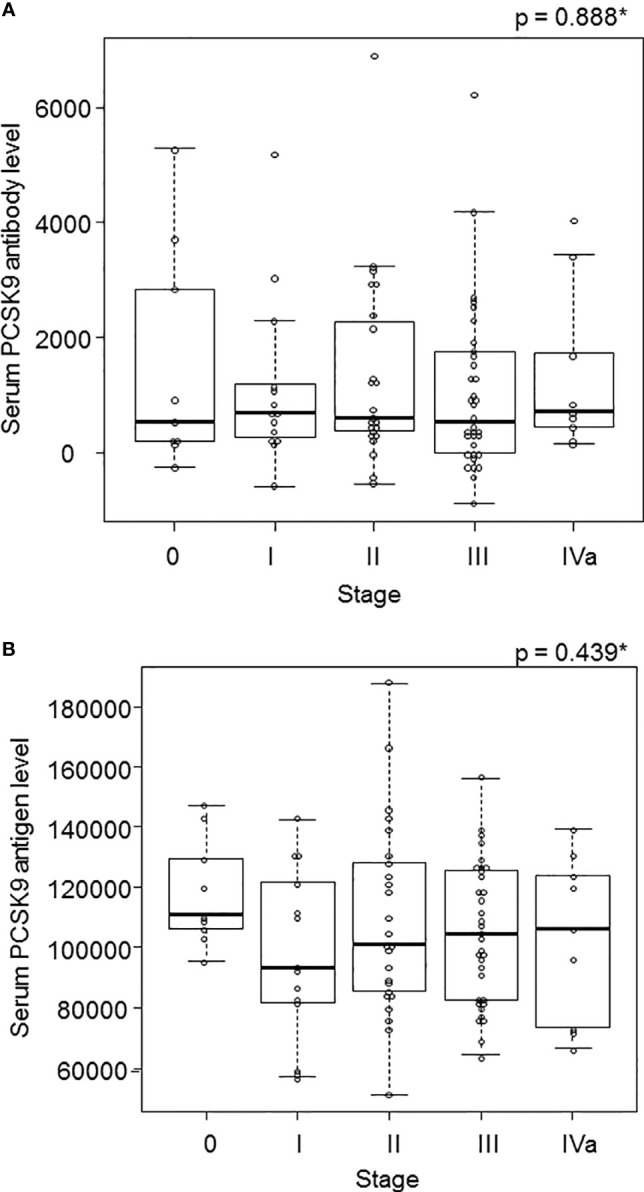
The s-PCSK9-Ab levels in different stages of esophageal cancer. The s-PCSK9-Ab levels examined by AlphaLISA are shown in box-whisker plots. Box bars represent 25, 50 and 75 percentiles. The upper and lower horizontal lines represent the limits. The dots present the deviant values **(A)**. The s-PCSK9-Ag level was also divided into each stage in esophageal cancer as well as PCSK9 antibody **(B)**. *p values calculated with Kruskal-Wallis test are shown.

Because the AUC showed the highest values for esophageal cancer among the solid tumors (**Figure 2**), we focused on the 91 surgical cases of esophageal cancer and examined the correlation between stages and s-PCSK9-Ab levels. The mean ± SD values of s-PCSK9-Ab levels for stage 0 (n = 9), stage I (n = 14), stage II (n = 25), stage III (n = 34), and stage IVa (n = 9) were 1518 ± 1934, 1142 ± 1472, 2602 ± 4804, 1037 ± 1429, and 1363 ± 1432, respectively (**Figure 5A**). No significant association was found between each stage and s-PCSK9-Ab level in the Kruskal-Wallis test. The level of s-PCSK9-Ag also showed no correlation with the stages. The mean ± SD values of s-PCSK9-Ag levels were as follows: stage 0, 118245 ± 18098, stage I, 97409 ± 28218; stage II, 109181 ± 31432; stage III, 105061 ± 22983; and stage IV, 103195 ± 27414 (**Figure 5B**). Of the 91 patients with esophageal cancer, 50 died within 5 years after surgery, with a mortality rate of 54.9%.

### Relationship Between S-PCSK9-Ab Levels and Overall Survival

**Figure 6:**
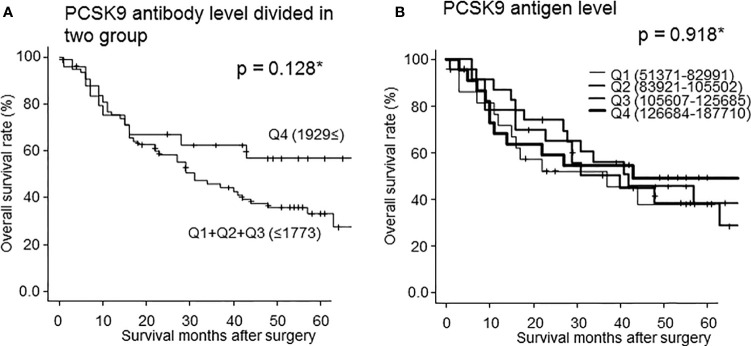
Comparison of overall survivals of the patients with esophageal cancer according to s-PCSK9-Ab levels classified into two groups (Q1+Q2+Q3 vs Q4) **(A)**. *Statistical analyses were performed by the Log-Rank test between two groups. The p value at 60 months after surgery was 0.128 **(A)**. The PCSK9 antigen level was also classified into every one-fourth quartiles according to antigen level **(B)**. The numbers in parentheses represent the antigen or antibody titers of each group. There was no statistic significant in each group.

To clarify the characteristics of s-PCSK9-Abs, we divided s-PCSK9-Ab levels into every one-fourth quartiles, Q1, Q2, Q3, and Q4 (**Figure 6A**). Serum anti-PCSK9 antibody levels of Q1 ranged from −887 to 219, those of Q2 ranged from 243 to 649, those of Q3 ranged from 660 to 1773, and those of Q4 ranged from 1929 to 22051. There was no significant survival difference between each group. However, the Q4 group showed a favorable prognosis compared with other groups. Although no statistically significant difference was observed between each group in the log-rank test (p = 0.128) (**Table 1**), the difference in survival rates between Q4 and Q1+Q2+Q3 groups increased by two times at 60 months after the surgery. We next calculated the PCSK9 antigen level and also classified the levels into one-fourth quartiles, in a similar manner as done for s-PCSK9-Ab level (**Figure 6B**). The levels of s-PCSK9-Ag of Q1 ranged from 51371 to 82991, those of Q2 ranged from 83921 to 105502, those of Q3 ranged from 105607 to 125685, and those of Q4 ranged from 126684 to 187710. No significant difference was observed between s-PCSK9-Ag levels and survival (p = 0.918).

**Table 1:** Univariate and multivariate analysis of risk factors for overall survival in the 91 patients with esophageal carcinoma.

	Univariate analysis	Multivariate analysis		
	p value^a^	Hazard ratio	95% CI	p value^b^
Sex	**0.046**	0.745	0.327-1.698	0.484
Male/Female				
Age	0.425			
>65/≤65				
Location	0.284			
Upper/Lower				
Tumor depth	**<0.001**	4.117	1.799-9.418	**<0.001**
T2-4/T0-1				
Lymph node metastasis	**<0.001**			
N+/N-				
SCC-Ag(ng/ml)	0.066	1.139	0.615-2.109	0.678
>1.5/≤1.5				
p53-Abs(U/ml)	0.063			
>1.30/≤1.30				
PCSK9-Ab	0.128	2.336	0.189-0.971	**0.042**
Q1Q2Q3 vs. Q4				
PD-L1	0.684			
>65.6/≤65.6				

N-, no lymph node metastasis; N+, lymph node metastasis exist; SCC-Ag, squamous cell carcinoma antigen; Abs, antibodies; PD-L1, programmed cell death ligand 1; CI, confidence interval; ^a^Log-rank test; ^b^Cox proportional hazard model.

Bold indicates a P-value of less than 0.05.

### Relationship Between Clinicopathological Factors and Prognosis

In the univariate analysis, no significant correlation was found between s-PCSK9-Ab levels and survival (**Table 1**, left panel) as mentioned in the previous section. We then performed Cox proportional hazards regression analysis (**Table 1**, right panel), with tumor depth, SCC-Ag, and PCSK9-Ab set as explanatory variables. The high s-PCSK9-Ab group (Q4) demonstrated a statistically significant correlation for survival (p = 0.042), whereas no significant difference was found between s-PCSK9-Ab levels and age, tumor location, SCC-Ag level, p53-Ab, and PD-L1 levels in the univariate analysis of survival.

### Relationship of High Serum PCSK9 Antibody Level With Clinicopathological Factors

Fisher’s exact probability test showed that high serum anti-PCSK9-Ab levels were significantly associated with high-PD-L1 levels (**Table 2**, left panel). However, sex, age, location, tumor depth, lymph node metastasis, SCC-Ag, and p53 antibody levels were not associated with serum PCSK9-Ab level. High-PD-L1 level (>65.6 pg/mL; according to our previous report) was also significantly associated with high serum PCSK9-Ab level in the logistic regression analysis (**Table 2**, right panel).

**Table 2:** Comparison of serum PCSK9 antibody levels according to clinicopathological characters of the patients with esophageal cancer.

Variables		Fisher’s exact probability test^a^			Logistic regression analysis^b^		
		PCSK9Q1+Q2+Q3	PCSK9Q4	p value	odds ratio	95% CI	p value
Sex	Male	52	18	0.784			
	Female	15	6				
Age	>65	41	12	0.348			
	≤65	26	12				
Location	Upper	11	3	0.753			
	Lower	56	21				
Tumor depth	T1	23	6	0.454			
	T2-T4	44	18				
Lymph node status	N0	26	15	0.057	0.489	0.163-1.470	0.202
	N1	41	9				
SCC-Ag(ng/ml)^c^	>1.5	21	10	0.305	1.390	0.449-4.280	0.569
	≤1.5	45	12				
p53-Abs(U/ml)^c^	>1.30	13	4	1.000			
	≤1.30	52	20				
PD-L1 (pg/ml)	>65.6	9	10	**0.006**	5.010	1.530-16.40	**0.008**
	≤65.6	52	12				

N0, no lymph node metastasis; N1, lymph node metastasis exist; SCC-Ag, squamous cell carcinoma antigen; Abs, antibodies; CI, confidence interval.

^a^Fisher’s exact probability test; ^b^Logistic regression analysis; ^c^Loss value.

Bold indicates a P-value of less than 0.05.

### Immunohistochemical Staining

**Figure 7:**
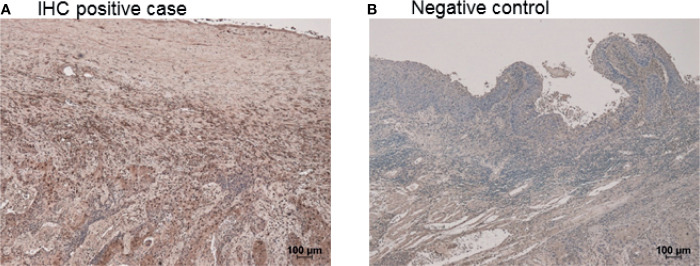
Immunohistchemical staining of esophageal cancer. Positively stained in the esophageal squamous cell cancer with PCSK9 antibody **(A)**. Cytoplasm and nuclear compartments of esophageal cancer was stained. In the negative control, esophageal squamous cell cancer was not stained without the primary PCSK9 antibody **(B)**.

In the immunohistochemical staining, the cytoplasm and nuclear compartments of esophageal squamous cell carcinoma tissue, but not the surrounding normal tissue, were predominantly stained with the PCSK9 antibody (**Figure 7A**). No apparent staining signal was observed in the negative control without the primary PCSK9 antibody (**Figure 7B**).

## Discussion

We demonstrated that s-PCSK9-Ab levels were significantly higher in patients with solid cancer than in HDs (**Figure 1**). Moreover, patients with high s-PCSK9-Ab levels were found to have a favorable prognosis in the case of esophageal cancer (**Figure 6A**). Although s-PCSK9-Ag levels were also elevated in patients with esophageal cancer (**Figure 4A**), they showed no association with prognosis (**Figure 6B**). This is the first report that s-PCSK9-Ab levels were examined by AlphaLISA which produced more stable and reproducible results than enzyme-linked immuno-sorbent assay (ELISA).

Regarding serum PCSK9 and cancers, low circulating levels of PCSK9 antigen independently predicted a better overall survival in non–small cell lung cancer (NSCLC) (40). Moreover, circulating levels of PCSK9 of <95 ng/mL during the second cycle of nivolumab treatment could independently predict a better overall survival in elderly patients with advanced, pretreated NSCLC (40). The assessment of serum PCSK9 might represent a useful tool for clinicians to evaluate and address patients with advanced NSCLC to the best therapeutic strategy (40). Momtazi-Borojeni et al. reported that PCSK9 inhibition may improve breast cancer (41), and that nanoliposomal anti-PCSK9 vaccine was useful for the treatment of colon and breast cancer (41, 42). It has been suggested that controlling serum PCSK9 level as a low titer contributes to the suppression of cancer development and improving the prognosis of cancer (21). These results are consistent with the concept that anti-PCSK9 autoantibodies have a tumor-suppressive role in a manner similar to that of anti-PCSK9 vaccine. Regarding the relationship between PCSK9 antigen and cancer, several reports have been published that it is better to keep the PCSK9 antigen titer low for improving prognosis, as mentioned above. Liu et al. reported that inhibition of PCSK9 can boost tumor response to immune checkpoint therapy, albeit through a mechanism independent of its cholesterol regulating functions. PCSK9 inhibition, either through genetic deletion or PCSK9 antibodies, caused a significant increase in tumor cell surface major histocompatibility protein class I (MHC I) expression, which promoted robust intratumoral infiltration of cytotoxic T-cells. Then, we focused on autoantibodies of PCSK9 and assumed that the higher the antibody titer, the better the prognosis by maintaining the PCSK9 antigen titer low.

In pathological and biochemical aspects, it has been reported that the mRNA and protein levels of PCSK9 were elevated in tumor tissues under glucose supply (43), as shown in **Figure 7**. In tumor-bearing mice, the transcriptional regulation of PCSK9 by glucose was found to enhance serum PCSK9 levels. In general, patients with cancer often show hyperglycemia due to various reasons (44), and the elevated PCSK9 expression increases the serum LDL levels, which can promote metastatic progression (17, 40, 44). Deng et al. reported the correlation between LDL and esophageal cancer prognosis (45). They discussed that LDL can enhance the growth and metastasis of esophageal cancer cells, which may be mediated by LDL receptor-related protein 1 (46, 47).

We also investigated the relationship between the antigen and antibody titers of PCSK9. We could not find any correlation in Spearman’s rank correlation analysis (**Figure 4D**). Their levels did not change with the progression of the cancer stage (**Figure 5A**). The absence of correlation of antigen and antibody was attributed to the PCSK9 protein assembled in the cancer tissue as detected by immunohistochemistry (**Figure 7**). And this time there was not shown statistically significant in PCSK9-Ab levels and sex, age, and pathological factors in univariate analysis (**Table 2**). As there was not confounding in these factors, and there was no report for serum PCSK9 antibody and cancer, we considered as follows. Antibody levels are increased by repeated leaking out of a small amount of antigens from cancer cells into the blood due to tissue destruction. Therefore, even if the amount of antigen is low, the antibody titer may be high. As IgG antibodies are highly stable in the blood, measuring antibody levels is highly sensitive and reproducible. To find out any function of serum PCSK9 antibody, we referred to Liu et al. journal (21). They reported that while administration of the anti-PCSK9 antibodies alone delayed tumor growth of MC38 tumors, their efficacies were enhanced significantly when combined with an anti-PD1 antibody, with long-term survival of some host mice (21). For that reason we also evaluated correlation between serum anti-PCSK9 antibody and PD-L1 levels.

Regarding biomarker, we recently reported about PD-L1, which is a poor prognostic biomarker in esophageal cancer (4). Unexpectedly, in the present study, PD-L1 correlated positively with favorable-prognosis-associated s-PCSK9-Ab levels (**Table 2**). To clarify the correlation of these two factors, we compared the characteristics in both poor prognosis cases. We selected the highest 23 cases of PD-L1 and the lowest 23 cases of s-PCSK9-Ab (Q1). We statistically evaluated the blood and biochemical relationship. Results showed that serum HbA1c level and total cholesterol level were higher in the low-PCSK9-Ab group than in the high-PD-L1 group (HbA1c; p = 0.022) (total cholesterol; p = 0.101) (**Supplementary Table S1**). This suggested that the poor prognosis of the low-PCSK9-Ab group but not of the high-PD-L1 group can be explained by hypercholesterolemia or diabetes mellitus because hyperlipidemia and diabetes mellitus have been reported to be relevant and independent risk factors for the recurrence of esophageal cancer (48).

Next, we evaluated the efficacy of measuring PCSK9 antibody levels. Among the lowest PD-L1 cases with a favorable prognosis, the PCSK9-Ab level was evaluated for the dead cases. The number of PD-L1 was selected at 23 cases (25%), because the prognosis was the most favorable with the lowest 25% of PD-L1 level according to our previous report (4). Of the 23 cases, 15 were dead. Among the 15 cases, 14 demonstrated low PCSK9-Ab levels (75 percentile or less). This result indicated that the mortality rate was high when the PCSK9-Ab levels were low despite low PD-L1 (**Supplementary Figure S1**). Therefore, consideration of s-PCSK9-Ab in addition to PD-L1 for predicting the overall survival is highly meaningful.

Currently, anti-PCSK9 monoclonal antibodies have been clinically applied worldwide for the therapy of hyperlipidemia to decrease LDL receptor levels on liver tissue (12, 13). This study has raised the possibility that anti-PCSK9 monoclonal antibody or PCSK9 vaccine is effective in improving the prognosis of low s-PCSK9-Ab patients with esophageal cancer. However, little is known about the function of PCSK9-Ab in cancer. Due to poor data on effectiveness and safety of PCSK9 inhibitors in cancer, the impact of PCSK9 inhibition in these pathological conditions is still unknown (P). Further study is required to determine the mechanism and the antitumor effect of anti-PCSK9 drugs in comparison with immune checkpoint inhibitor therapy.

## Conclusion

The levels of s-PCSK9-Ab were higher in patients with solid cancers such as esophageal cancer, gastric cancer, colorectal cancer, lung cancer, and breast cancer than in HDs. Regarding esophageal cancer, patients with high s-PCSK9-Ab levels showed favorable prognosis compared with those with low s-PCSK9-Ab levels.

## Data Availability Statement

The original contributions presented in the study are included in the article/**Supplementary Material**. Further inquiries can be directed to the corresponding author.

## Ethics Statement

The studies involving human participants were reviewed and approved by Ethics Committee of Toho University, Graduate School of Medicine (nos. A19033) and Chiba University Graduate School of Medicine (No. 2018-320) (Japan). The patients/participants provided their written informed consent to participate in this study.

## Author Contributions

All authors contributed to the article and approved the submitted version. MI and TH: project development and research, manuscript writing, and editing. HS, MT, KY and HT: manuscript editing. YO, SY, TS, TN, MS, FS and KF: collecting serum sample. SY-L, YI, HY and BJ: sample measurement.

## Funding

This research was supported by the Project for Cancer Research and Therapeutic Evolution (P-CREATE) from the Japan Agency for Medical Research and Development, AMED and supported by JSPS KAKENHI Grant Number 16K10520, 15K10117, 20K07810 and 19K09451. This work was partly supported by a research grant of Toho University School of Medicine.

## Conflict of Interest

The authors declare that the research was conducted in the absence of any commercial or financial relationships that could be construed as a potential conflict of interest.

## Publisher’s Note

All claims expressed in this article are solely those of the authors and do not necessarily represent those of their affiliated organizations, or those of the publisher, the editors and the reviewers. Any product that may be evaluated in this article, or claim that may be made by its manufacturer, is not guaranteed or endorsed by the publisher.
