# Supplementary material for: Association of Serum Anti-PCSK9 Antibody Levels with Favorable Postoperative Prognosis in Esophageal Cancer
Source: Front Oncol. 2021 Aug 24;11:708039. doi: 10.3389/fonc.2021.708039 (PMC8421770; doi:10.3389/fonc.2021.708039)
Supplement: Supplementary file 1 [file DataSheet_1.pdf]

Figure

Supplementary FIGURE S1 | Relationship of s-PCSK9-Ab levels and poor prognosis.

In the lowest 23 cases of PD-L1 with favorable prognosis, 15 cases were dead. s-PCSK9-Ab levels of the 15 patients with poor prognosis are shown.

Supplementary Figure S1.

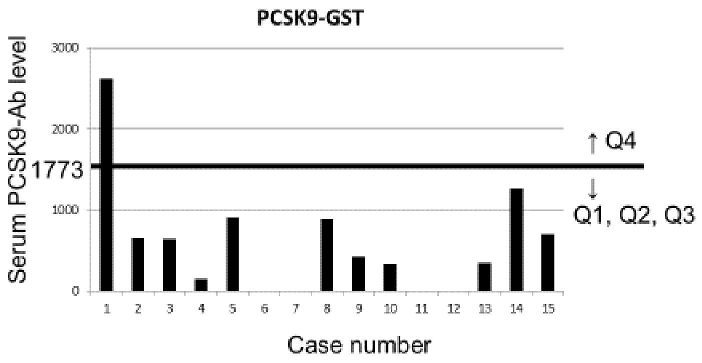

Tables

Supplementary Table S1 Comparison of low s-PCSK9-Ab 23 cases and high PD-L1 23 cases according to clinical blood and biochemical examination of the patients with esophageal cancer.

Variables	Low PCSK9-Ab (n=23)	High PD-L1 (n=23)	p value
alive/dead	10/13	10/13	
Total Cholesterol (mg/dl)	187 (126-278)	172 (111-322)	0.101
Albumin (g/dl)	3.8 (2.4-4.7)	3.6 (2.6-4.5)	0.316
HbA1C (%)	5.7 (4.1-7.9)	5.3 (4.8-5.7)	***0.022***
C reactive protein (mg/dl)	0.25 (0.0-0.9)	0.30 (0.0-3.0)	0.393
White blood cell	5800 (3800-12100)	6600 (3400-11900)	0.956
Hemoglobin (g/dl)	12.9 (7.1-16.2)	13.0 (8.2-15.3)	0.878
Platelet (10*4)	25.0 (15.2-48.5)	24.2 (7.7-44.0)	0.339

HbA1c, hemoglobin A1c; Ab, antibody;

*Mann-Whitney U Test
